# Leptin as a mediator of tumor-stromal interactions promotes breast cancer stem cell activity

**DOI:** 10.18632/oncotarget.6014

**Published:** 2015-10-27

**Authors:** Cinzia Giordano, Francesca Chemi, Salvatore Panza, Ines Barone, Daniela Bonofiglio, Marilena Lanzino, Angela Cordella, Antonella Campana, Adnan Hashim, Pietro Rizza, Antonella Leggio, Balázs Győrffy, Bruno M. Simões, Robert B. Clarke, Alessandro Weisz, Stefania Catalano, Sebastiano Andò

**Affiliations:** ^1^ Centro Sanitario, University of Calabria, Arcavacata di Rende, Italy; ^2^ Department of Pharmacy, Health and Nutritional Sciences, University of Calabria, Arcavacata di Rende, Italy; ^3^ IRCCS SDN (Istituto di Ricerca Diagnostica e Nucleare), Napoli, Italy; ^4^ Laboratory of Molecular Medicine and Genomics, Department of Medicine and Surgery, University of Salerno, Baronissi, Italy; ^5^ Norwegian Centre for Molecular Medicine (NCMM), University of Oslo, Oslo, Norway; ^6^ Breast Cancer Now Research Unit, Institute of Cancer Sciences, University Manchester, Manchester, UK; ^7^ MTA TTK Lendület Cancer Biomarker Research Group, Budapest, Hungary; ^8^ 2nd Dept. of Pediatrics, Semmelweis University, Budapest, Hungary; ^9^ MTA-SE Pediatrics and Nephrology Research Group, Budapest, Hungary

**Keywords:** breast cancer, leptin, microenvironment, CAFs, breast cancer stem cells

## Abstract

Breast cancer stem cells (BCSCs) play crucial roles in tumor initiation, metastasis and therapeutic resistance. A strict dependency between BCSCs and stromal cell components of tumor microenvironment exists. Thus, novel therapeutic strategies aimed to target the crosstalk between activated microenvironment and BCSCs have the potential to improve clinical outcome. Here, we investigated how leptin, as a mediator of tumor-stromal interactions, may affect BCSC activity using patient-derived samples (*n* = 16) and breast cancer cell lines, and determined the potential benefit of targeting leptin signaling in these model systems. Conditioned media (CM) from cancer-associated fibroblasts and breast adipocytes significantly increased mammosphere formation in breast cancer cells and depletion of leptin from CM completely abrogated this effect. Mammosphere cultures exhibited increased leptin receptor (*OBR*) expression and leptin exposure enhanced mammosphere formation. Microarray analyses revealed a similar expression profile of genes involved in stem cell biology among mammospheres treated with CM and leptin. Interestingly, leptin increased mammosphere formation in metastatic breast cancers and expression of *OBR* as well as *HSP90*, a target of leptin signaling, were directly correlated with mammosphere formation in metastatic samples (*r* = 0.68/*p* = 0.05; *r* = 0.71/*p* = 0.036, respectively). Kaplan–Meier survival curves indicated that *OBR* and *HSP90* expression were associated with reduced overall survival in breast cancer patients (HR = 1.9/*p* = 0.022; HR = 2.2/*p* = 0.00017, respectively). Furthermore, blocking leptin signaling by using a full leptin receptor antagonist significantly reduced mammosphere formation in breast cancer cell lines and patient-derived samples. Our results suggest that leptin/leptin receptor signaling may represent a potential therapeutic target that can block the stromal-tumor interactions driving BCSC-mediated disease progression.

## INTRODUCTION

Carcinoma of the breast is the most common malignancy and the leading cause of cancer-related death in women worldwide [1]. Despite improvements in diagnosis and treatment, metastatic or recurrent disease and resistance to therapy remain the principal causes of death for breast cancer patients.

In the last years, multiple reports have shown that a subpopulation of cancer cells displaying stem cell properties and named as cancer stem cells (CSCs) plays a crucial role in sustaining tumor growth and progression. These cells are characterized by their ability to undergo self-renewal, a process that drives tumorigenesis, and to differentiate into the non-self-renewing cells forming the tumor bulk [2, 3]. From a clinical point of view, the main concern with CSCs is related to their resistance to conventional treatments (e.g. endocrine-, chemo- and radio-therapy), a feature that might be the underlying cause of tumor recurrence and metastases [4–6]. Similar to embryonic and somatic stem cells, the self-renewal and differentiation of CSCs are regulated by both intrinsic and extrinsic pathways whose dysregulation may be a key event initiating carcinogenesis. Among the intrinsic pathways, an important role is displayed by developmental signals such as Wnt, Hedgehog, Janus kinase 2-signal transducer and activator of transcription 3 (JAK2-STAT3) and Notch pathways that are frequently deranged in cancers [7]. Extrinsic signals that regulate stem cell behaviour originate in the surrounding stem cell microenvironment, termed as cancer stem niche. This niche contains a number of cell types, including mesenchymal stem cells (MSCs), cancer-associated fibroblasts (CAFs), adipocytes, endothelial and immune cells, all of which, through networks of cytokines and growth factors, have been shown to influence tumor growth and metastasis [8]. Thus, strategies aimed to specifically target the interaction between CSCs and their microenvironment may represent an important approach to improve patient outcome.

Adipocytes and CAFs are the major components in breast cancer microenvironment, and along with their secreted factors represent key players in stroma-epithelial cell interactions. As an important paracrine mediator, the adipocyte-derived cytokine leptin, that we have recently demonstrated to be also secreted by CAFs [9], has been correlated with breast cancer occurrence. Leptin exerts its biologic function through binding to its receptor (OBR) which activates multiple downstream signaling pathways such as those involving JAK2-STAT3, mitogen-activated protein kinase (MAPK), and phosphatidylinositol 3-kinase/protein kinase B (PI3K/AKT) [10]. Leptin and both short and long OBR isoforms are overexpressed in breast cancer, especially in higher grade tumors and are associated with distant metastases [11, 12]. It has been extensively demonstrated that this cytokine, acting in an autocrine, endocrine and paracrine manner, may modulate many aspects of breast tumorigenesis from initiation and primary tumor growth to metastatic progression [13–15]. Besides, crosstalk with other different signaling molecules such as estrogens, growth factors and inflammatory cytokines further increases leptin impact on breast tumor progression [16–21]. Moreover, leptin is able to shape the tumor microenvironment within the mammary gland by inducing multiple concurrent events such as migration of endothelial cells, angiogenesis and recruitment of macrophages and monocytes [13–15, 22]. Interestingly, recent studies have also reported that leptin signaling may be involved in the promotion of CSC phenotype [23–25] and that inhibition of STAT3 suppresses leptin-induced CSC activity and cancer progression in diet-induced obese rats [26].

The aim of the current study was to evaluate the role of leptin, as a mediator of the tumor-stroma interaction, in regulating breast CSC activity using breast cancer cell lines and patient-derived breast cancer cells isolated from metastatic ascites and pleural effusions. Particularly, we investigated: i) the impact of CAFs and adipocytes isolated from stromal breast tissues on mammosphere formation and self-renewal in breast cancer cells; ii) the specific role of leptin and its receptor in influencing breast CSC phenotype in the context of the tumor microenvironment; iii) the effect of inhibiting leptin signaling as potential therapeutic target to reduce breast CSC activity in *in vitro* and *ex vivo* models.

## RESULTS

### CAFs and adipocytes induce mammosphere formation in breast cancer cells through leptin secretion

**Figure 1:**
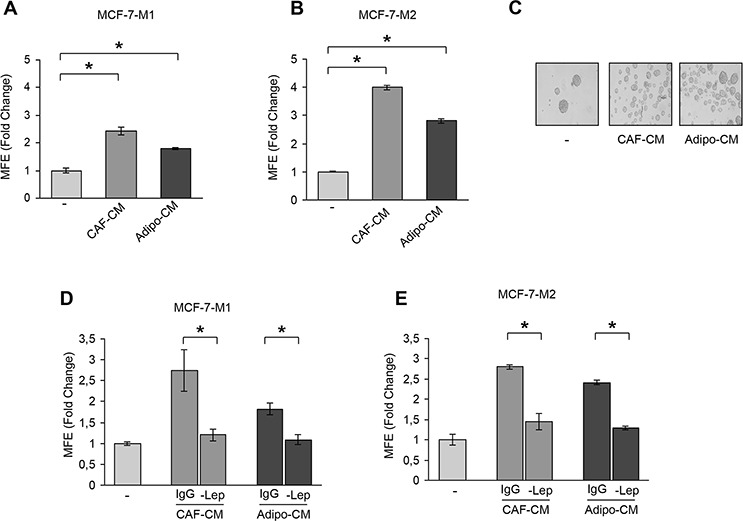
Leptin mediates the effects of stromal cell-CM on breast cancer cell mammosphere formation Mammosphere Forming Efficiency (MFE) evaluated in MCF-7-M1 (**A**) and MCF-7-M2 (**B**) in the presence or absence (−) of CAF- and Adipocyte-derived Conditioned Media (CAF-CM and Adipo-CM, respectively). MFE was calculated by dividing the number of mammospheres (colonies > 50 μm) formed by the number of the cells plated and expressed as fold change compared to untreated cells (−). (**C**) Representative phase-contrast images of mammospheres treated as in panel (B) are shown. MFE evaluated in MCF-7-M1 (**D**) and MCF-7-M2 (**E**) in the presence or absence (−) of leptin-immunodepleted CAF-CM and Adipo-CM (-Lep). IgG: CM immunodepleted with nonspecific antibody. The values represent the means ± s.d. of three different experiments each performed in triplicate. *p < 0.05.

To assess the ability of stromal cells to affect CSC activity in breast cancer cells we performed co-culture experiments. As experimental models for breast CSCs (BCSCs), we used estrogen receptor (ER)-α-positive MCF-7 cells grown as mammospheres. This culture system has been used to characterize, enrich and propagate breast cancer cells with stem-like phenotype, relying on the feature of stem cells to escape anoikis and grow as spheroids in anchorage-independent conditions [27]. MCF-7 mammosphere cells were characterized by flow cytometric analysis that revealed an enrichment of CD44^+^/CD24^−^ subpopulation compared to MCF-7 monolayer cells (Supplementary Figure S1A). In addition, real-time PCR further revealed that genes associated with stem cell phenotype, including *OCT4, N-CAD, BMI1, SOX4*, were expressed in mammosphere cells at higher levels than in monolayer cells (Supplementary Figure S1B). Moreover, MCF-7 mammosphere cells were also analyzed for the expression of ERα (Supplementary Figure S1C and 1D). As stromal cells, we used either CAFs isolated from biopsies of primary breast tumors or human breast adipocytes obtained after preadipocyte differentiation. CAFs possessed the basic fibroblast characteristics with long and spindle-shaped morphology and highly expressed alpha-smooth muscle actin (α-SMA), vimentin, and fibroblast activation protein (*FAP*) (Supplementary Figure 2A and 2B). Adipocytes displayed a classical morphological phenotype characterized by accumulation of lipid droplets associated with the expression of specific markers as PPARγ and leptin (*OB*) (Supplementary Figure S2C). Using co-culture experiments, we examined mammosphere formation from MCF-7 cells in the presence or absence of conditioned media (CM) harvested from CAFs and adipocytes. Compared to the cells cultured alone, MCF-7 cells co-cultured with CAF- or adipocyte-derived CM showed a significant enhancement in mammosphere forming efficiency (MFE) (Figure 1A). Stem cells are maintained in the primary mammospheres through self-renewal, and are able to give rise to secondary mammospheres when cells from the primary spheres are dissociated and allowed to grow in anchorage-independent conditions. Therefore, we carried out secondary mammosphere cultures to examine the effects of CM on BCSC self-renewal. Our experiments demonstrated an increased self-renewal in MCF-7 cells treated with CAF- and adipocyte-derived CM in the first generation compared with the untreated spheres (Figure 1B and 1C). These data suggest that BCSC activity is influenced by soluble factors secreted from stromal cells. Thus, given the role of leptin as an important cytokine secreted by both CAFs and adipocytes, we assessed the impact of leptin in the context of the heterotypic signaling working in BCSC–stromal interactions. First, ELISA measurement in CM from stromal cells showed that leptin levels were 2,4 ± 0,12 ng/mg protein and 20,32 ± 2 ng/mg protein in CAF- and adipocyte-derived CM, respectively. Leptin was then immunodepleted from CAF- and adipocyte-derived CM using a specific leptin antibody, and resulting media were tested for the ability to induce mammosphere formation in breast cancer cells. AS shown in Figure 1D and 1E, leptin depletion significantly decreased the MFE/self-renewal promoted by stromal cell-derived CM.

### Targeting leptin signaling reduces stem cell activity mediated by stromal cells

**Figure 2:**
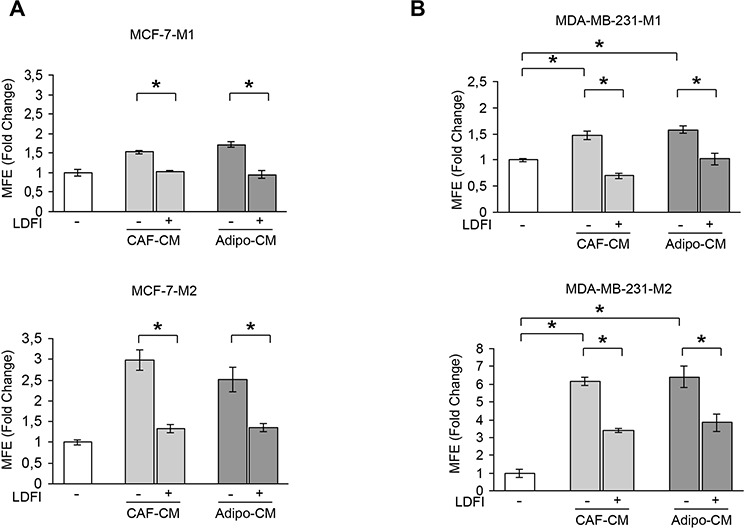
Effects of a selective leptin receptor antagonist on breast cancer stem cell activity MFE evaluated in MCF-7-M1 and MCF-7-M2 (**A**) and in MDA-MB-231-M1 and MDA-MB-231-M2 (**B**) treated with CAF-CM and Adipo-CM with/without peptide LDFI (1 μg/ml). The values represent the means ± s.d. of three different experiments each performed in triplicate. *p < 0.05.

Our previous experiments indicate that leptin may represent an important paracrine molecule that mediates the interaction between stromal cells and BCSCs. To support this observation, we tested the effect of a full leptin receptor antagonist, peptide LDFI, on BCSC activity. We have previously shown that this peptide inhibits leptin-induced breast cancer growth *in vitro* and exhibits anti-neoplastic activities *in vivo* [28]. Our data demonstrated that treatment with peptide LDFI significantly reduced MFE/self-renewal promoted by stromal cell-derived CM in MCF-7 cells (Figure 2A). To extend the results obtained, we have grown the ERα-negative MDA-MB-231 breast cancer cells as mammospheres and evaluated the effects of CAF- or adipocyte-CM in the presence or absence of peptide LDFI. Treatment of MDA-MB-231 mammosphere cultures with CAF- or adipocyte-derived CM significantly increased MFE/self-renewal and the addition of the OBR antagonist LDFI strongly reduced these effects (Figure 2B), confirming that leptin/leptin receptor may play a crucial role in maintaining the BCSC traits mediated by stromal cells in different cellular backgrounds.

### Leptin signaling regulates mammosphere formation/self-renewal activity of breast cancer cells

**Figure 3:**
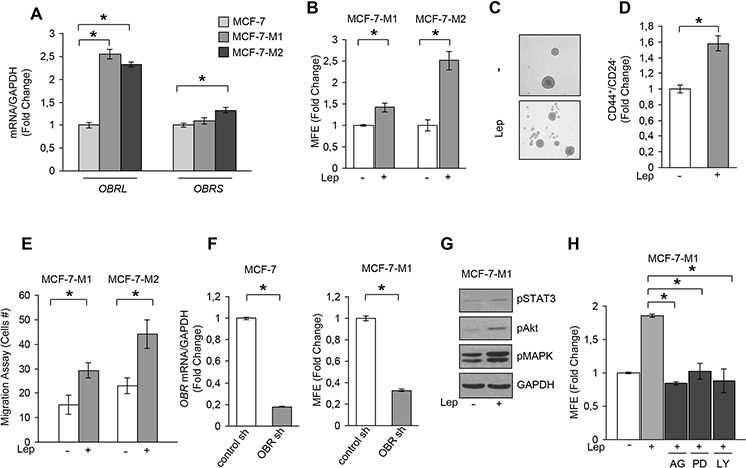
Leptin induces MFE in breast cancer cells **A.** Leptin receptor long (*OBRL*) and short (*OBRS*) isoform mRNA levels, evaluated by real time RT-PCR, in MCF-7, MCF-7-M1 and MCF-7-M2 cells. Each sample was normalized to its *GAPDH* mRNA content. **B.** MFE in MCF-7-M1 and MCF-7-M2 in the presence or absence (−) of leptin 500 ng/ml (Lep). **C.** Representative phase-contrast images of mammospheres treated as in panel (B) are shown. **D.** CD44^+^/CD24^−^ population in MCF-7-M2 cells treated or not (−) with Lep. **E.** Transmigration assays in MCF-7-M1 and MCF-7-M2-derived cells treated or not (−) with Lep. **F.** MCF-7 cells were stably transfected with either a scrambled shRNA (control-sh) or OBR shRNA (OBR-sh). *OBRL* mRNA content was evaluated by real time RT-PCR (left panel). Each sample was normalized to its *GAPDH* mRNA content. MFE in MCF-7-M1 derived from either control-sh or OBR-sh clones (right panel). **G.** Immunoblotting of phosphorylated (p), STAT3 (Tyr^705^), Akt (Ser^473^), and MAPK (Thr^202^/Tyr^204^) at the indicated residues measured in cellular extracts from MCF-7-M1 cells treated or not (−) with Lep. GAPDH, loading control. **H.** MFE in MCF-7-M1 treated with Lep and AG490 (AG-20 μmol/L), PD98059 (PD-10 μmol/L) or LY294002 (LY-10 μmol/L). The values represent the means ± s.d. of three different experiments each performed in triplicate. *p < 0.05.

Having shown that stromal cells regulate BCSC activity through secretion of leptin, we next investigated the direct involvement of this cytokine in the regulation of mammosphere formation/self-renewal in MCF-7 cells. In agreement with previous data demonstrating that leptin receptor plays a crucial role in maintaining cancers in a stem cell-like state [23–26], we found that MCF-7 mammosphere cultures exhibited increased *OBR* mRNA expression and in a greater extent the long isoform, compared to monolayer cells (Figure 3A). Accordingly, leptin treatment of mammosphere cultures resulted in a significant increase in MFE/self-renewal and in an enhanced percentage of CD44^+^/CD24^−^ population compared with untreated cells (Figure 3B, 3C and 3D). Accordingly, in MDA-MB-231 mammosphere cultures, we observed a significant increase in the long isoform of *OBR* mRNA expression compared to monolayer cells, and an enhanced MFE/self-renewal after leptin exposure (Supplementary Figure S3), demonstrating that this cytokine can directly regulate BCSC activity.

Since BCSCs display increased cell motility and invasion, we tested the effects of leptin on the migratory potential of MCF-7 mammospheres. Our data clearly showed that leptin exposure increased the number of migrated cells suggesting that this cytokine can facilitate the invasive behavior of BCSCs (Figure 3E). Next, *OBR* expression was stably knocked-down using lentiviral delivered short hairpin RNA (*OBR* sh) in MCF-7 cells (Figure 3F, left panel). Suppression of *OBR* expression led to a significant inhibition of MFE (Figure 3F, right panel), implying that this gene is necessary for maintaining cancer stem-like properties in breast cancer cells. In addition, we observed that leptin treatment induced the phosphorylation of specific OBR downstream signaling molecules such as STAT3, Akt and p42/44 MAPK (Figure 3G). As expected, the increase in MFE induced by leptin was reversed by the JAK2-STAT3 inhibitor AG490, the MEK1 inhibitor PD98059 and the PI3K/AKT inhibitor LY294002 (Figure 3H), suggesting that leptin promotes stem cell properties *via* activation of classical leptin signaling pathways. In agreement with these observations, we also found an up-regulation of well-known leptin target genes as *OBR* and the heat shock protein 90 (HSP90) [20] in MCF-7 cells treated with leptin (Supplementary Figure S4A and 4B)

### Gene expression profiling in leptin or stromal CM-treated mammosphere-derived cells

**Figure 4:**
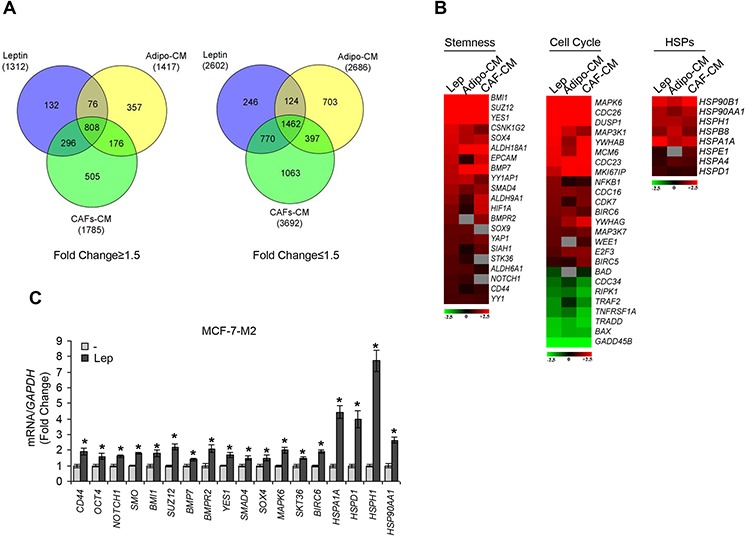
Gene expression profiling in mammosphere cultures treated with stromal cell-CM or leptin **A.** Venn diagram of up-(left panel) and down-(right panel) regulated transcript identified by microarray analysis in MCF-7-M2 cells treated with CAF-CM, Adipo-CM or Lep compared to untreated samples. **B.** Heat-maps of stemness related-genes, cell cycle related-genes and HSP family genes from microarray data. Gene expression changes were calculated in treated cells with respect to the untreated controls. Transcript showing a DiffScore ≤ − 30 and ≥ 30, corresponding to a *p*-value of 0.001, and significant fold change in treated vs untreated ≥ 1.5 were considered. **C.** Real-time RT-PCR validation of a subset of genes in MCF-7-M2 cells treated or not (−) with Lep. Each sample was normalized to its *GAPDH* mRNA content. The values represent the means ± s.d. of three different experiments each performed in triplicate. *p < 0.05 *vs* untreated (−) sample.

To determine whether leptin, CAF- and adipocyte-CM may similarly affect gene expression profile in mammosphere-derived cells, we performed gene expression profiling analysis on total RNA extracted from the second generation spheres. Microarray results highlighted several RNAs differentially expressed in treated *vs* untreated MCF-7 mammospheres. Venn diagram analysis was used to compare the gene lists and to identify those genes that are unique and in common among the three treatments (Figure 4A). A total of 2270 transcripts were commonly regulated in all treated samples (808 up- and 1462 down-regulated transcripts, respectively). It should be noted that the global overlap among genes expressed in treated samples includes a number of genes known to play a role in stem cell biology such as *BMI1*, *SUZ12*, *YES1*, *SOX4* (Figure 4B, left panel, Supplementary Table S2). Similar trends were also observed for the expression of other genes involved in cell cycle control (Figure 4B, middle panel, Supplementary Table S3). Moreover, treated samples displayed up-regulation of some transcripts related to the heat shock protein family, that recently have been suggested to be crucial in sustaining proliferation and self-renewal of stem cells [29] (Figure 4B, right panel, Supplementary Table S4). To validate our microarray study MCF-7 mammospheres treated with leptin were evaluated for the expression of a panel of genes by using real-time PCR (Figure 4C). Taken together, gene expression profile analyses strongly support the role of leptin as a crucial paracrine molecule able to mediate the microenvironment effects on BCSC activity.

### Leptin increases patient-derived mammosphere formation/self-renewal activity

**Figure 5:**
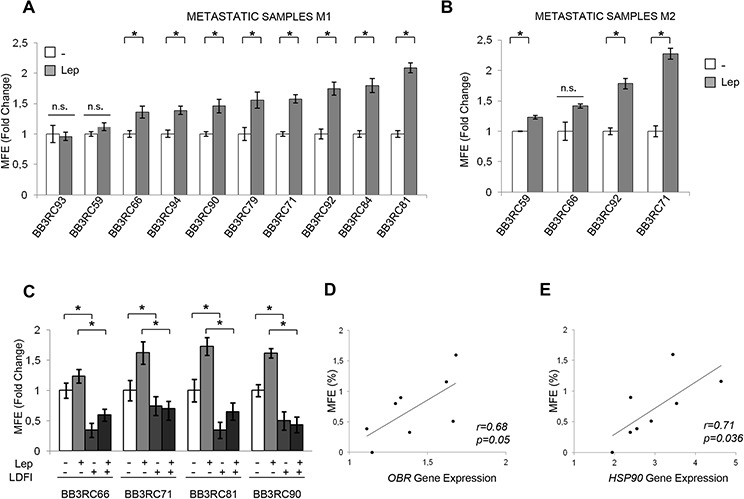
Leptin enhances mammospheres formation/self-renewal activity in patient-derived metastatic cells 10 metastatic fluid samples obtained from breast cancer patients (BB3RC59/BB3RC66/BB3RC71–94) undergoing palliative drainage of symptomatic ascites or pleural effusions were used (Table 1). MFE in metastatic patient-derived cells grown as primary (Metastatic samples M1) (**A**) or secondary (Metastatic samples M2) (**B**) mammospheres in the presence or absence (−) of Lep. (**C**) MFE in 4 Metastatic sample M1 untreated (−) or treated with Lep, peptide LDFI (1 μg/ml), and Lep+LDFI. The values represent the means ± s.d. of three different experiments each performed in triplicate. *p < 0.05. n.s.:nonsignificant. Correlation between *OBR* (**D**) or *HSP90* mRNA expression (**E**) in cells of the metastatic fluids and MFE (8 patients/BB3RC29–70) (Pearson correlation coefficient, *r* = 0.68, *p* = 0.05; *r* = 0.71, *p* = 0.036, respectively).

The role of leptin in the regulation of BCSC activity was then evaluated by using patient-derived breast cancer cells isolated from metastatic ascites or pleural effusions. Tumor histology, grade, hormone receptors and HER2 status of the primary tumors were reported in Table 1. Mammosphere cultures treated with leptin resulted in a significant increase in MFE compared to untreated samples (*n* = 10, Figure 5A). Secondary mammosphere formation was observed only in four samples and treatment with leptin significantly increased self-renewal in three of them (Figure 5B). Besides, four human metastatic samples taken from patients with breast cancer were also treated with peptide LDFI. MFE induced by leptin was significantly decreased with the addition of LDFI (Figure 5C). Interestingly, treatment with peptide LDFI alone reduced the mammosphere formation, underlying how this peptide negatively interferes with leptin autocrine loop (Figure 5C). Then, to investigate the direct involvement of OBR in the regulation of mammosphere formation, *OBR* gene expression was analyzed in cells from metastatic ascites and pleural effusion fluids using microarray data. There was a significant direct correlation between the expression of *OBR* mRNA in cells from the metastatic fluids and MFE (*r* = 0.68; *p* = 0.05, Figure 5D). In agreement with the microarray data obtained in MCF-7 mammospheres, a significant correlation between MFE and *HSP90* gene expression in the same metastatic patient-derived samples (*r* = 0.71; *p* = 0.036) was also observed (Figure 5E). These data suggest that patients with higher levels of *OBR* and *HSP90* mRNAs in cells of metastatic fluids have greater *ex vivo* CSC activity.

**Table 1:** Summary of metastatic patients-derived cancers

SAMPLE ID	AGE	SOURCE	Histology	GRADE	ER	PR	HER2
BB3RC29	70	ASC	UN	UN	POS	POS	NEG
BB3RC46	68	ASC	ILC	2	POS	POS	NEG
BB3RC50	46	ASC	IDC	2	POS	POS	NEG
BB3RC59^1^	69	ASC	ILC	2	POS	POS	NEG
BB3RC60	66	ASC	ILC	2	POS	POS	NEG
BB3RC65^2^	62	ASC	ILC	2	POS	POS	NEG
BB3RC66^1^	69	ASC	ILC	2	POS	POS	NEG
BB3RC70^2^	62	ASC	ILC	2	POS	POS	NEG
BB3RC71	48	PE	UN	3	POS	POS	POS
BB3RC79	UN	PE	IDC	3	NEG	NEG	NEG
BB3RC81	55	ASC	IDC	2	POS	POS	NEG
BB3RC84	UN	PE	UN	3	NEG	NEG	NEG
BB3RC90	66	PE	IDC/ILC	2	P0S	POS	NEG
BB3RC92	61	ASC	IDC	1	POS	POS	NEG
BB3RC93	UN	ASC	UN	UN	POS	POS	NEG
BB3RC94	41	ASC	UN	UN	POS	POS	NEG

1, 2 These samples were obtained at different time points from the same patients

Sixteen patient-derived breast cancer samples were used in this study. Tumor histology and grade for metastatic samples (ASC and PE) relates to the primary cancer. Abbreviation: **UN** unknown, **PE** pleural effusion, **ASC** ascites sample, **ILC** invasive lobular carcinoma, **IDC** invasive ductal carcinoma, **POS** positive, **NEG** negative, **ER** Estrogen Receptor, **PR** Progesterone Receptor, **HER2**, epidermal growth factor receptor 2.

### OBR expression correlates with reduced overall survival in breast carcinomas

**Figure 6:**
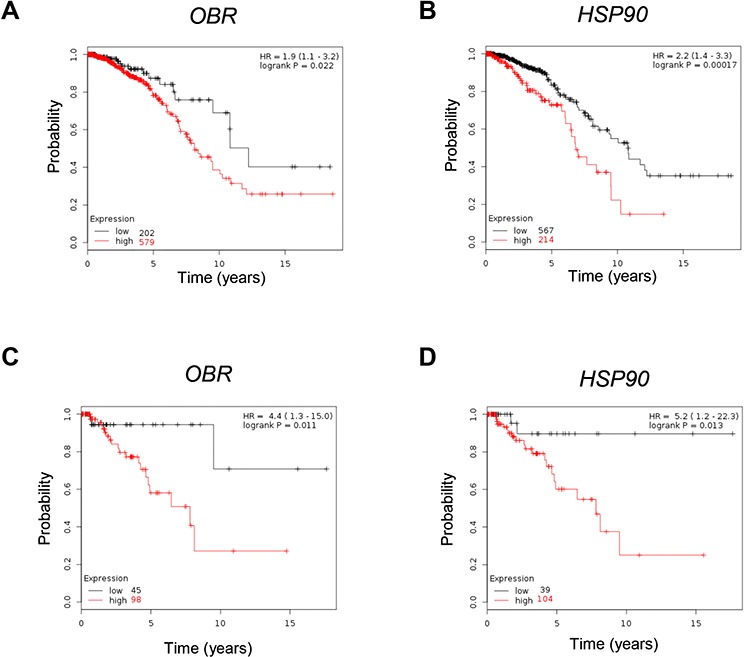
Correlation between *OBR* and *HSP90* mRNA levels and overall survival in breast cancer Kaplan–Meier survival analysis in breast carcinoma patients (*n* = 781) with high and low *OBR* (**A**) or *HSP90* (**B**) expression analyzed as described in *Materials and Methods*. Kaplan–Meier survival analysis in basal breast cancer patients (*n* = 143) with high and low *OBR* (**C**) or *HSP90* (**D**) expression. Kaplan-Meier survival graph, and hazard ratio (HR) with 95% confidence intervals and logrank *P* value.

To investigate the clinical significance of *OBR* gene expression in human breast cancers the relationship between *OBR* levels and overall survival (OS) of breast cancer patients (*n* = 781) was estimated by Kaplan–Meier analysis. Survival curves indicated that women with high *OBR* expression exhibited a lower rate of OS than those with low *OBR* expression (HR = 1.9, *p* = 0.022) (Figure 6A). Similarly, breast carcinoma patients with high *HSP90* expression had decreased OS compared with those with low *HSP90* expression (*HR = 2.2, p = 0.00017*) (Figure 6B).

Basal-like breast cancer is an aggressive tumor subtype, composed by primitive undifferentiated cells. Indeed, basal-like breast tumors, which are enriched for CD44^+^/CD24^−^ cells, exhibit epithelial–mesenchymal transition features and express high levels of stem cell-regulatory genes [30–34]. In agreement with these observations, the results of the Kaplan-Meier analysis indicated a more relevant discrimination in terms of overall survival between high and low expression of *OBR* and *HSP90* in basal breast cancer patients (*n* = 143) (*HR = 4.4*, *p = 0.011; HR = 5.2, p = 0.013* respectively) (Figure 6C and 6D).

## DISCUSSION

The heterotypic signals arising in the tumor-associated stroma have been shown to be important in inducing and maintaining a stem-like state in the tumor cells through either the secretion of soluble molecules or cell–cell communication [35, 36]. Particularly, in the case of breast carcinoma, it has been reported that various types of stromal cells *via* growth factors and cytokines may enhance the proliferation and survival of BCSCs, induce angiogenesis, and recruit tumor-associated macrophages and other immune cells, which in turn secrete additional factors promoting tumor cell invasion and metastasis [8].

Here we demonstrated, for the first time, that leptin and its receptor play a crucial role in mediating the interaction between stromal cells (CAFs and adipocytes) and BCSCs. The initial conditioned media experiments indicated that the entire complement of secretory proteins released by CAFs and adipocytes significantly increase MFE/self-renewal in breast cancer cells. An important role is played by leptin as a fundamental environmental regulator of CSCs in the cancer stem niche. Indeed, either leptin immunodepletion from CAF- and adipocyte-derived CM or inhibition of leptin signaling by using peptide LDFI, a small-molecule that acts as a full leptin receptor antagonist [28], reduced the effects of CM on mammosphere formation. Gene expression profiling revealed a significant overlap of regulated genes in mammosphere cells following treatment with CAF-, adipocyte-derived CM or leptin. Of particular interest was the observation that genes commonly expressed in all treated-samples include several of those involved in stemness. Among these, the polycomb gene *BMI1*, which has been reported to play an important role in self-renewal of stem cells and has a positive correlation with clinical grade/stage and poor prognosis [37], was one of the most highly induced in all treated cells. One of the features of CSCs is the uncontrolled proliferation, perhaps due to a reduced responsiveness to negative growth regulators or to the loss of contact inhibition and gap junction intercellular communication [38]. Our results clearly evidenced that a number of genes involved in cell cycle control showed a similar expression profile upon treatment with stromal-CM and leptin. Another family of genes, crucial in sustaining self-renewal of stem cells [29], is the heat shock protein family. We have previously demonstrated that the HSP90, a main functional component of this chaperone complex, is a target of leptin in breast cancer cells [20]. Our microarray data showed that some transcripts of the HSP family were upregulated in stromal-CM and leptin-treated samples. Thus, since the expression pattern of genes regulated by leptin and involved in stem cell biology closely mirrors those modulated by stromal cells, it is reasonable to speculate that leptin may represent a critical paracrine molecule in mediating the microenvironment effects on BCSC activity.

The expression of the leptin receptor is a characteristic feature of CSCs and of a broad array of embryonic and induced pluripotent stem cells, which exhibit an increased response to leptin including phosphorylation and activation of STAT3 and induction of stem cell markers, as *OCT4* and *SOX2* [23]. Leptin receptor has also been reported as a marker for identification and *in vivo* fate of bone marrow mesenchymal stem cells (MSCs) [39] and leptin signaling represents an essential step for the enhanced survival, chemotaxis and therapeutic properties of MSCs induced by hypoxia [40, 41]. Moreover, it has been reported that leptin is able to regulate and activate several signaling pathways and oncogenes which are critically implicated in BCSCs [42–46] and leptin deficiency in MMTV-Wnt-1 transgenic mice results in functional depletion of BCSCs leading to less tumor outgrowth [24]. More recently, it has also been demonstrated that OBR is necessary for maintaining a CSC-like state in TNBC cells [25] and high OBR expression induced by the adiposity-leptin enriched environment generates a population with enhanced CSC properties and tumorigenic capacity [26]. Our studies extended these previous findings by demonstrating a direct involvement of leptin in sustaining breast cancer stem cell behavior using both breast cancer cell lines and metastatic breast cancer patient-derived cells. We found that MCF-7 mammospheres exhibited increased *OBR* mRNA expression, while OBR silencing caused a significant reduction in the sphere-forming efficiency. Treatment with leptin induced an increase in MFE, self-renewal and an enhanced percentage of CD44^+^/CD24^−^ cell population, through the activation of the classical signaling pathways. Importantly, we also showed that leptin is able to increase the mammosphere formation and self-renewal activity in metastatic breast cancer cells isolated from patients. Moreover, *OBR* mRNA expression, analyzed in cells from metastatic fluids, was directly correlated with mammosphere formation activity *ex vivo*. In agreement with our data of gene expression profile, a significant positive correlation between MFE and *HSP90* mRNA expression in the same metastatic patient-derived samples was observed.

It has been previously reported that high-grade tumors associated with poor prognosis display an enrichment of BCSCs [47, 48]. Here, using Kaplan-Meier analysis we found that *OBR* expression, which is crucial in maintaining stem cell phenotype, was associated with reduced overall survival in breast carcinomas suggesting its potential role as a prognostic factor. Interestingly, in basal-like breast cancer patients, a more relevant discrimination in terms of overall survival between high and low *OBR* expression could be observed. Finally, we demonstrated that blocking leptin signaling by using the peptide LDFI significantly reduced mammosphere formation in metastatic breast cancer patient-derived cells, suggesting that strategies aimed at inhibiting leptin signaling represent a rationale therapeutic approach to target cancer stem cells.

In conclusion, our findings identify, for the first time, leptin as an important paracrine molecule that mediates the interaction between stromal cells and BCSCs, providing novel insights into understanding how BCSCs are influenced by the tumor microenvironment. As clinical implications, these data suggest that targeting leptin/leptin receptor signaling generated in the microenvironment may be useful for BCSC eradication and eventually to prevent recurrence and metastasis in patients with breast carcinoma.

## MATERIALS AND METHODS

### Cell culture

Human MCF-7 and MDA-MB-231 breast cancer epithelial cells were acquired in 2010 and 2015 respectively, from American Type Culture Collection where they were authenticated, stored according to supplier's instructions, and used within 4 months after frozen aliquots recovery. Breast subcutaneous human female preadipocytes (Lot.#:BR071812B; BR070810) were from Zen-Bio. Adipocytes, obtained following differentiation procedure, were routinely maintained in Adipocyte maintenance medium (Zen-Bio). Every 4 months, cells were authenticated by single tandem repeat analysis at our Sequencing Core; morphology, doubling times, estrogen sensitivity, and mycoplasma negativity were tested (MycoAlert, Lonza).

### Cancer associated fibroblast (CAF) isolation

Human breast cancer specimens were collected in 2013 from primary tumors of patients who signed informed consent following the procedures previously described [9]. Briefly, small pieces of fresh tumor excision were digested (500 IU collagenase in Hank's balanced salt solution; Sigma; 37°C for 2 h). After differential centrifugation (90 g for 2 min), the supernatant containing CAFs was centrifuged (500 g for 8 min), resuspended, and cultured in RPMI-1640 medium supplemented with 15% FBS and antibiotics. The fibroblastic nature of the isolated cells was confirmed by microscopic determination of morphology, and characterization by αSMA, vimentin, pan-Cytokeratin and fibroblast activation protein (FAP) expression. CAFs between 4 and 10 passages were used.

### Immunofluorescence

Immunofluorescence assay was performed as described [9] using anti-α-SMA or ERα antibodies and fluorescein isothiocyanate-conjugated secondary antibody (Santa Cruz Biothecnology).

### Conditioned medium (CM) and leptin-immunodepleted CM

CM from CAFs and adipocytes and leptin-immunodepleted CM were obtained as described [9]. Leptin levels were measured by ELISA (LDN).

### Metastatic breast cancer patient-derived cells

Pleural effusion and ascites samples were obtained from patients with metastatic breast cancer undergoing palliative drainage at The Christie Hospital NHS Foundation Trust Manchester (UK). Metastatic breast sample details in Table 1. Ascites and pleural effusions were centrifuged at 1000 g for 10 min at 4^°^C and suspended in PBS. Erythrocytes and leucocytes were removed by centrifugation through Lymphoprep solution (Axis Shield), followed by removal of CD45-positive cells using anti-CD45 magnetic beads (Miltenyi Biotec). Single cell suspension of breast cancer epithelial cells was then used to perform mammosphere assay.

### Mammosphere culture

MCF-7 and MDA-MB-231 monolayer cells were enzymatically and manually disaggregated to obtain single-cell suspension. Single cells were plated in ultralow attachment plates (Corning) at a density of 500 cells/cm^2^ in a serum-free Human mammary epithelial cell growth medium (HUMEC), supplemented with B27, 20 ng/mL human epidermal growth factor (EGF), 4 μg/mL heparin, 5 μg/ml insulin, 1 ng/ml hydrocortisone, 1 mg/ml penicillin-streptomycin and 0,25 μg/ml amphotericin B (Life Technologies). Growth factors and treatments (leptin, Life Technologies; AG490 Sigma; PD98059/LY294002 Calbiochem) were added to the mammosphere cultures every 3 days. After 7 days mammospheres > 50 μm (primary mammospheres-M1) were counted using a microscope (x40 magnification), collected, enzymatically dissociated, plated at the same seeding density used in the primary generation to obtain secondary mammospheres-M2. Mammosphere cultures from metastatic breast patient-derived cells was assessed as described [49]. Mammospheres forming efficiency (MFE) was calculated as number of mammospheres per well/number of cells seeded per well and reported as fold versus control.

### Flow cytometry

Mammospheres were dispersed to obtain single-cell suspension. Cells were washed in PBS with 2,5% BSA and stained with FITC anti-human CD44 and PE anti-human CD24 (BD Biosciences), according to the supplier's protocol. Flow cytometric analysis was performed on a FACScan and acquisition was performed with WinDI software (Becton Dickinson).

### Reverse transcription and real-time reverse transcriptase PCR assays

*PPARγ/OB*/*FAP/36B4* mRNA expression was evaluated by the RT–PCR method as described [50]. Real-time RT-PCR was assessed using SYBR Green Universal PCR Master Mix (Biorad). Each sample was normalized on its *GAPDH* mRNA content. Relative gene expression levels were calculated as previously described [50]. Primers in Supplementary Table S1.

### Immunoblot analysis

Protein extracts were subjected to SDS-PAGE as described [50]. Immunoblots show a single representative of 3 separate experiments.

### Transmigration assays

Mammosphere derived MCF-7 cells were placed in the upper compartments of Boyden chamber (8-μm membranes/Corning Costar) and transmigration assay was performed as described [9].

### Lentiviral transfection

We established stable OBR sh MCF-7 cell line using the lentiviral expression system (GeneCopoeia; lentiviral plasmid sh-clone #HSH010584). 48 h after transfection with packaging plasmids and pLentiviral plasmids of target gene in HEK293 cells, supernatants containing lentiviral particles were filtered (0.45 μm PES), mixed with polybrene (8 μg/ml) and used to infect MCF-7 cells. 24 h after infection, cells were selected with 2 μg/mL puromycin overtime to eliminate un-infected cells. *OBR* mRNA expression in stable MCF-7 clones was evaluated by real-time RT-PCR.

### Microarray and data analysis

Microarray analyses were carried out on total RNA from MCF-7-M2 mammosphere-derived cells treated with CAF-CM, Adipocyte-CM or Leptin by pooling equal amounts of nucleic acids extracted from three independent cell cultures. Gene expression profiling was performed in triplicate using 500 ng of each RNA pool as described [51], cRNAs were hybridized for 18 h at 55°C on Illumina HumanHT-12 v4.0 BeadChips (Illumina Inc.) and scanned with an Illumina iSCAN. Data analyses were performed with GenomeStudio software version 2011.1 (Illumina Inc.). Data were normalized with the quantile algorithm and genes were considered if the detection *p*-value was < 0.01. Statistical significance was calculated with the Illumina DiffScore, a proprietary algorithm that uses the bead standard deviation to build an error model. Transcripts showing a DiffScore ≤− 30 and ≥ 30, corresponding to a *p*-value of 0.001 and significant fold change in treated *vs* untreated ≥ 1.5 were considered. Venn diagram was generated using Venny 2.0 software. Heat-maps were generated with the Multiexperiment Viewer 4.9 software after performing one way hierarchical clustering of transcripts with the average linkage method and Euclidian distance.

Raw microarray data have been deposited, in a format complying with the Minimum Information About a Microarray Gene Experiment (MIAME) guidelines of the Microarray Gene Expression Data Society (MGED), in the EBI ArrayExpress database (http://www.ebi.ac.uk/arrayexpress) with Accession Number: E-MTAB-3641.

Total RNA from 8 different metastatic breast cancer samples was extracted using the RNeasy Plus Mini Kit (QIAGEN). The Exon Gene Array ST1 platform (Affimetrix) was used to assess gene expression. Data obtained were analysed using Bioconductor R Software. The mean of log2 gene expression values was calculated across all 8 patient derived samples for each individual gene.

### Construction of RNA-seq database

RNA-seq data was obtained from the TCGA depository. We transferred the pre-processed level 3 data generated by the Illumina HiSeq 2000 RNA Sequencing Version 2 platform. Expression levels for these samples were computed using a combination of MapSplice and RSEM. Individual patient files were merged into a single database using the plyr R package [52].

### Statistical analyses

Each datum point represents the mean ± s.d. of three different experiments. Data were analyzed by Student's *t* test using the GraphPad Prism 4 software. *P* < 0.05 was considered as statistically significant. Pearson correlation coefficient (r) was used to measure the correlation between *OBR* or Heat Shock Protein 90 (*HSP90*) gene expression of 8 metastatic breast cancer samples and mean MFE; a 2-tailed *p* ≤ 0.05 was considered statistically significant.

Kaplan-Meier analysis was performed as described [53]. Kaplan-Meier survival graph, and hazard ratio with 95% confidence intervals and logrank *P* value were calculated and plotted in R using Bioconductor packages.

## SUPPLEMENTARY FIGURES AND TABLES


