# Supplementary material for: Leptin as a mediator of tumor-stromal interactions promotes breast cancer stem cell activity
Source: Oncotarget. 2015 Oct 27;7(2):1262–75. doi: 10.18632/oncotarget.6014 (PMC4811458; doi:10.18632/oncotarget.6014)
Supplement: Supplementary file 1 [file oncotarget-07-1262-s001.pdf]

SUPPLEMENTARY FIGURES AND TABLES

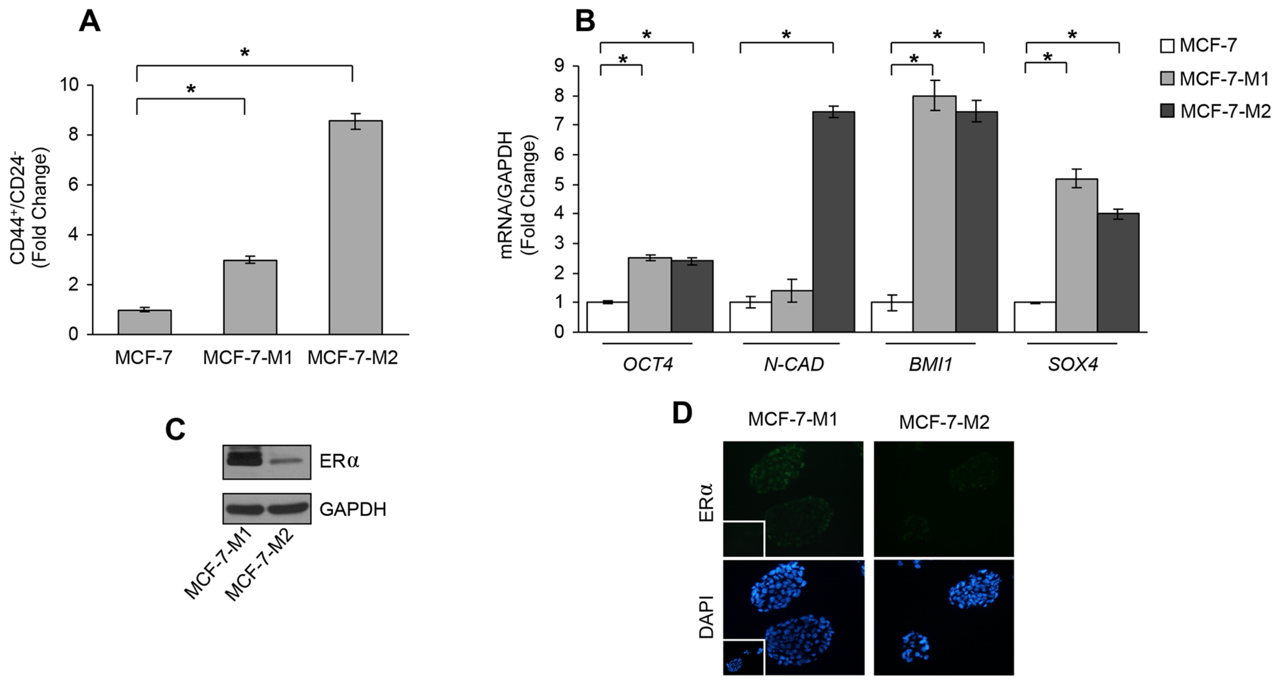

Supplementary Figure S1: A. Flow cytometric analysis of CD44+/CD24− phenotype in MCF-7 monolayer cells, and in MCF-7 cells grown as primary- (MCF-7-M1) and secondary-mammospheres (MCF-7-M2). The values represent the means ± s.d. of three different experiments each performed in triplicate. *$p < 0.05$. **B.** *OCT4*, *N-CAD, BMI1* and *SOX4* mRNA content evaluated by real-time RT–PCR in MCF-7, MCF-7-M1 and MCF-7-M2 cells. Each sample was normalized to its *GAPDH* mRNA content. The values represent the means ± s.d. of three different experiments each performed in triplicate. *$p < 0.05$. **C.** Immunoblotting for estrogen receptor (ER)-α in MCF-7-M1 and MCF-7-M2 cells. GAPDH as loading control. **D.** Immunofluorescence of ERα and DAPI staining (for nuclei detection) in MCF-7-M1 and MCF-7-M2 cells. Small squares, negative control.

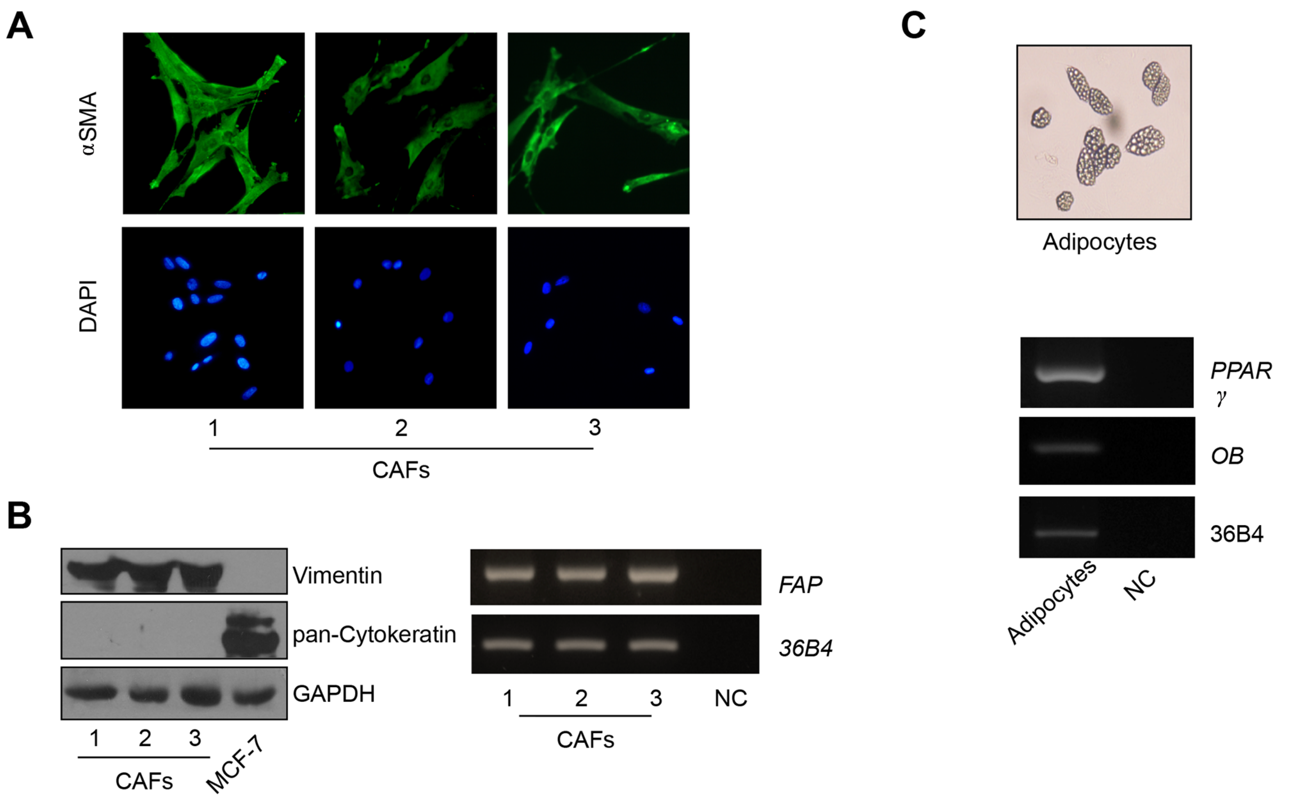

Supplementary Figure S2: A. Cancer Associated Fibroblasts (CAFs) were isolated from primary breast tumour biopsies by collagenase digestion. Immunofluorescence of alpha-smooth muscle actin (α-SMA) and DAPI staining (for nuclei detection). **B.** *Left panel, i*mmunoblotting of Vimentin and pan-Cytokeratin protein expression, GAPDH as loading control. *Rigth panel*, RT-PCR analysis of Fibroblast Activation Protein (*FAP*) mRNA levels. **C.** Subcutaneous preadipocytes from breast tissues were stimulated to differentiate into mature adipocytes. *Upper panel*, a typical bright-field picture of adipocytes characterized by accumulation of lipid droplets; *lower panel*, RT-PCR analysis, for expression of the specific adipocyte markers *PPARγ* and leptin (*OB*). *36B4* (internal standard). NC, negative control.

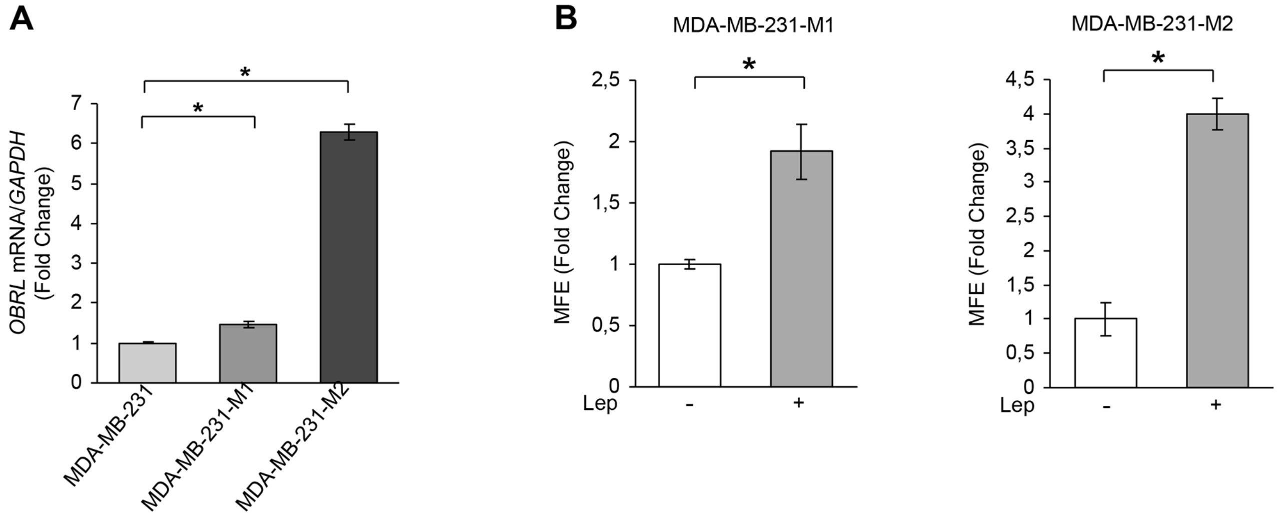

Supplementary Figure S3: A. Leptin receptor long (*OBRL)* mRNA content evaluated by real-time RT–PCR in MDA-MB-231, MDA-MB-231-M1 and MDA-MB-231-M2 cells. Each sample was normalized to its *GAPDH* mRNA content. **B.** MFE evaluated in MDA-MB-231-M1 and MDA-MB-231-M2 cells in the presence or absence (−) of leptin 500 ng/ml (Lep) in the mammosphere culture media. The values represent the means ± s.d. of three different experiments each performed in triplicate. *$p < 0.05$.

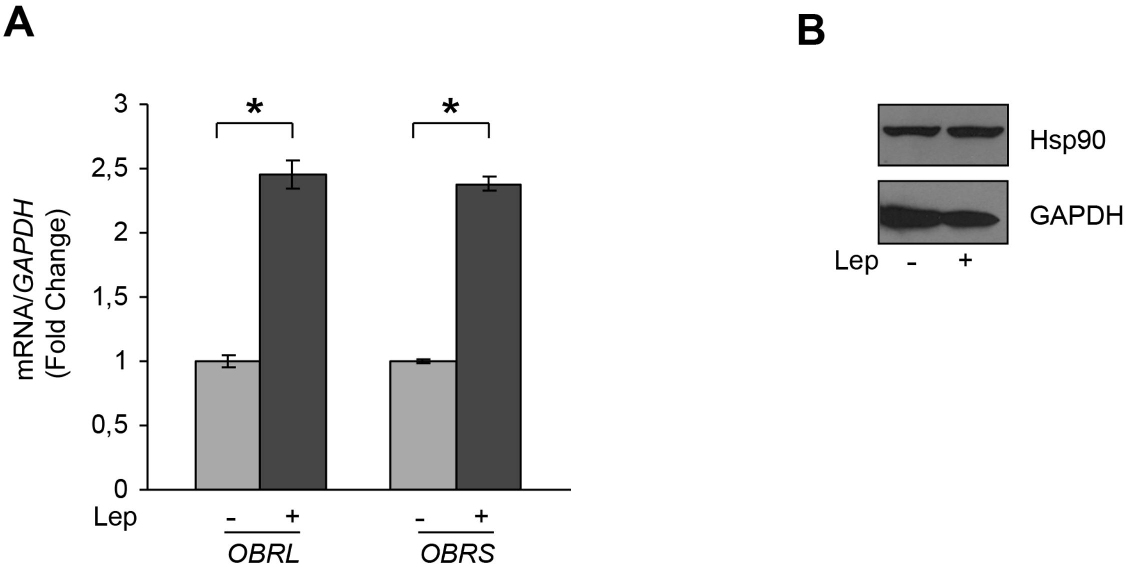

Supplementary Figure S4: A. Leptin receptor long (*OBRL)* and short *(OBRS)* mRNA content evaluated by real-time RT–PCR in MCF-7-M1 cells. Each sample was normalized to its *GAPDH* mRNA content. The values represent the means ± s.d. of three different experiments each performed in triplicate. $*p < 0.05$. **B.** Immunoblotting analysis of HSP90 levels in total protein extracts from MCF-7-M1 cells in the presence or absence (−) of leptin 500 ng/ml (Lep) in the mammosphere culture media. GAPDH was used as loading control.

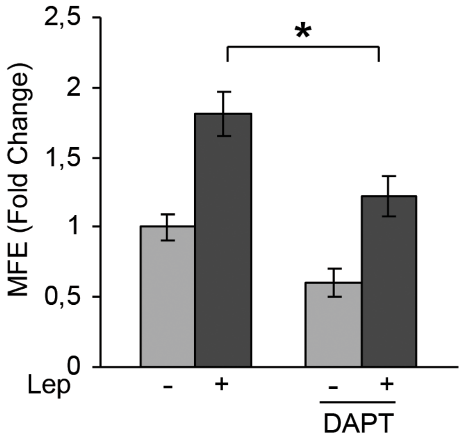

Supplementary Figure S5: MFE evaluated in MCF-7-M1 cells treated with leptin 500 ng/ml (Lep) in the presence or absence (−) of DAPT (5 μM) in the mammosphere culture media. The values represent the means ± s.d. of three different experiments each performed in triplicate. $*p < 0.05$.

Supplementary Table S1: Oligonucleotide primers used in this study

Gene symbol	Gene name	Primer sequences
PPARγ	Peroxisome Proliferator-Activated Receptor gamma	For 5′-GAGTTCATGCTTGTCAAGGATGC-3′<br>Rev 5′-CGATATCACTGGAGATCTCGCC-3′
OB	Leptin	For 5′ -GAGACCTCCTCCATGTGCTG-3′<br>Rev 5′- TGAGCTCAGATATCGGGCTGAAC-3′
FAP	Fibroblast activation protein	For 5′-AGAAAGCAGAACTGGATGG-3′<br>Rev 5′-ACACACTTCTTGCTTGGAGGAT-3′
36B4	36B4	For 5′-CTCAACATCTCCCCCTTCTC-3′<br>Rev 5′- CAAATCCCATATCCTCGT -3′
GAPDH	glyceraldehyde-3-phosphate dehydrogenase	For 5′- CCCACTCCTCCACCTTTGAC-3′<br>Rev 5′- TGTTGCTGTAGCCAAATTCGTT-3′
OBRL	Leptin Receptor Long Isoform	For 5′-GATAGAGGCCCAGGCATTTTTTA-3′<br>Rev 5′- CACCACTCTCTCTCTTTTTGATTGA-3′
OBRS	Leptin Receptor Short Isoform	For 5′-ATTGTGCCAGTAATTATTTCCTCTTCC-3′<br>Rev 5′-CCACCATATGTTAACTCTCAGAAGTTCA-3′
CD44	CD44 molecule	For 5′- CCTTTGATGGACCAATTACCATAAC-3′<br>Rev 5′- TCAGGATTCGTTCTGTATTCTCCTT-3′
OCT4	POU class 5 homeobox 1 (POU5F1)	For 5′- AGCGACTATGCACAACGAGA-3′<br>Rev 5′- CCATAGCCTGGGTACCAAA-3′
N-CAD	N-cadherin	For 5′- ACAGTGGCCACCTACAAAGG-3′<br>Rev 5′- CCGAGATGGGGTTGATAATG-3′
NOTCH1	Notch 1	For 5′-GTGACTGCTCCCTCAACTTCAAT-3′<br>Rev 5′-CTGTCACAGTGGCCGTCACT-3′
SMO	Smoothened, frizzled family receptor	For 5′- CACCCTGGCCACATTCGT-3′<br>Rev 5′- CGCATTGACGTAGAAGAGAATAACA-3′
BMI1	Polycomb ring finger oncogene	For 5′- GTGCTTTGTGGAGGGTACTTCAT-3′<br>Rev 5′-TACACGTTTTACAGAAGGAATGTAGAC-3′
SUZ12	SUZ12 polycomb repressive complex 2 subunit	For 5′- AGCTTACGTTTACTGGTTTCTTCCA-3′<br>Rev 5′- GCAAACTTTCACAAGCAGGACTT-3′
BMP7	Bone morphogenetic protein 7	For 5′-GGGAACGCTTCGACAATGAG-3′<br>Rev 5′- CGATTCCCTGCCCAAGTG-3′
BMPR2	Bone morphogenetic protein receptor, type II	For 5′- GCCTTTGGGAGAAATCAAAAGGGG-3′<br>Rev 5′- CATTCTGAATTGAGGGAGGAGTGG-3′
YES1	v-yes-1 Yamaguchi sarcoma viral oncogene homolog 1	For 5′- CCTCGAGAATCTTTGCGACTAGA-3′<br>Rev 5′-CCATTCCATGTTCCCATCCA-3′
SMAD4	SMAD family member 4	For 5′-GGAGCTCATCCTAGTAAATG-3′<br>Rev 5′-GACGGGCATAGATCACATGA-3′
SOX4	SRY (sex determining region Y)-box 4	For 5′- GGCCTCGAGCTGGGAATCGC-3′<br>Rev 5′- GCCCACTCGGGGTCTTGCAC-3′
MAPK6	Mitogen-activated protein kinase 6	For 5′-GTCGGAGAAGTCCCGTTGTATC-3′<br>Rev 5′-TCCAGCTCACCACAATCACAAT-3′
SKT36	Serine/threonine kinase 36	For 5′- CGCATCCTACACCGAGATATGA-3′<br>Rev 5′- GCAAATCCAAAGTCACAGAGCTT-3′

(*Continued*)

Gene symbol	Gene name	Primer sequences
BIRC6	Baculoviral IAP repeat-containing protein 6	For 5′- GGACCACCGCATCTCTACAT -3′<br>Rev 5′- GCAGTGGATGAAGCCAGCCT-3′
HSPA1A	Heat shock 70kDa protein 1A	For 5′- CCTGTGTTTGCAATGTTGAAATTT -3′<br>Rev 5′- CTCTGCATGTAGAAACCGGAAA-3′
HSPD1	Heat shock 60kDa protein 1 (chaperonin)	For 5′-GATGTTGATGGAGAAGCTCTAAGTACA-3′<br>Rev 5′-TGCCACAACCTGAAGACCAA-3′
HSPH1	Heat shock 105kDa/110kDa protein 1	For 5′-AAATCAGCAAATCACTCATGCAA-3′<br>Rev 5′-ATGCTCGGCCATGAAATCTT-3′
HSP90AA1	Heat shock protein 90kDa alpha, class A member 1	For 5′-ATTGCCCAGTTGATGTCATTGA-3′<br>Rev 5′-ATGCATCTGATGAATTTGAAATGAG-3′

Supplementary Table S2: Selection of relevant modulated genes involved in stem cell biology in MCF-7-M2 derived cells

Gene symbol	Gene name	Lep	Adipo-CM	CAF-CM
BMI1	Polycomb ring finger oncogene	+4.10	+3.63	+5.23
SUZ12	SUZ12 polycomb repressive complex 2 subunit	+3.43	+3.25	+3.73
YES1	v-yes-1 Yamaguchi sarcoma viral oncogene homolog 1	+3.04	+3.34	+3.51
CSNK1G2	Casein kinase 1, gamma 2	+2.32	+2.05	+1.70
SOX4	SRY (sex determining region Y)-box 4	+2.28	+2.03	+2.15
ALDH18A1	Aldehyde dehydrogenase 18 family, member A1	+2.24	+2.51	+3.24
EPCAM	Epithelial cell adhesion molecule	+2,15	+1,28	+2.23
BMP7	Bone morphogenetic protein 7	+2.10	+3.11	+3.26
YY1AP1	YY1 associated protein 1	+2.07	+2.12	+1.68
SMAD4	SMAD family member 4	+1.97	+1.67	+1.9
ALDH9A1	Aldehyde dehydrogenase 9 family, member A1	+1.88	+1.36	+2.20
HIF1A	Hypoxia-inducible Factor 1, alpha subunit	+1,74	+1.23	+2.18
BMPR2	Bone morphogenetic protein receptor, type II	+1.69	ns	+1.82
SOX9	SRY (sex determining region Y)-box 9	+1.59	+1.59	ns
YAP1	Yes-associated protein 1	+1.58	+1.54	+1.72
SIAH1	Siah E3 ubiquitin protein ligase 1	+1.58	+1.17	+1.55
STK36	Serine/threonine kinase 36	+1.50	+1.27	ns
ALDH6A1	Aldehyde dehydrogenase 6 family, member A1	+1.50	+1.48	+1.24
NOTCH1	Notch 1	+1.44	+1.35	ns
CD44	CD44 molecule (Indian blood group)	+1.44	+1.16	+1.33
YY1	YY1 transcription factor	+1.42	+1.43	+1.40

Numbers represent the ratio of the values of treated compared with untreated MCF-7 M2 spheres obtained by microarray data analysis. Statistical significance was calculated with the Illumina DiffScore, only genes with a DiffScore ≤− 30 and ≥ 30, corresponding to a *p*-value of 0.001, were considered as statistically significant. ns: nonsignificant.

Supplementary Table S3: Selection of relevant modulated genes involved in cell cycle control in MCF-7-M2 derived cells

Gene symbol	Gene name	Lep	Adipo-CM	CAF-CM
MAPK6	Mitogen-activated protein kinase 6	+6.27	+6.27	+7.90
CDC26	Cell division cycle 26 homolog (S. cerevisiae)	+3.85	+5	+4.8
DUSP1	Dual specificity protein phosphatase 1	+2.74	+2.71	+2.5
MAP3K1	Mitogen-activated protein kinase kinase kinase 1	+2.55	+2.19	+1.9
YWHAB	Tyrosine 3-monooxygenase/ tryptophan 5-monooxygenase act pro, b	+2.46	+1,86	+2.67
MCM6	Michromosome maintance complex componeny 6	+2,42	+1.95	+3.44
CDC23	Cell division cycle 23 homolog (S. cerevisiae)	+2.41	+2.77	+3.64
MKI67IP	MKI67 (FHA domain) interacting nucleolar phosphoprotein	+2.14	+1.69	+2.46
NFKB1	Nuclear factor of kappa light polypeptide gene enhancer in B-cells 1	+1.81	+1.21	+1.31
CDC16	Cell division cycle 16 homolog (S. cerevisiae)	+1,79	+1.8	+1.7
CDK7	Cyclin-dependent kinase 7	+1.75	+1.3	+1.74
BIRC6	Baculoviral IAP repeat-containing protein 6	+1.72	+1.78	+1.53
YWHAG	Tyrosine 3-monooxygenase/ tryptophan 5-monooxygenase act pro, g	+1.64	+1.97	+2.33
MAP3K7	Mitogen-activated protein kinase kinase kinase 7	+1.59	+1.55	+1.68
WEE1	WEE1 homolog (S. pombe)	+1.59	ns	+1.41
E2F3	Transcription factor	+1.50	+1.8	+1.82
BIRC5	Baculoviral IAP repeat-containing protein 5	+1.32	+1.27	+1.75
BAD	BCL2-antagonist of cell death	-1.44	ns	-1.23
CDC34	Cell division cycle 34 homolog (S. cerevisiae)	-1.64	-1.36	-1.84
RIPK1	Receptor (TNFRSF)-interacting serine-threonine kinase 1	-1.73	-1.65	-2.06
TRAF2	TNF receptor-associated factor 2	-1.85	-1.24	-1.82

(*Continued*)

Gene symbol	Gene name	Lep	Adipo-CM	CAF-CM
TNFRSF1A	Tumor necrosis factor receptor superfamily, member 1A	-1.85	-1.67	-2.16
TRADD	TNFRSF1A-associated via death domain	-2.18	-2.04	-2.24
BAX	BCL2-associated X protein	-2.21	-2.05	-2.07
GADD45B	Growth arrest and DNA-damage-inducible beta	-2.29	-2.05	-2.69

Numbers represent the ratio of the values of treated compared with untreated MCF-7 M2 spheres obtained by microarray data analysis. Statistical significance was calculated with the Illumina DiffScore, only genes with a DiffScore ≤− 30 and ≥ 30, corresponding to a *p*-value of 0.001, were considered as statistically significant. ns: nonsignificant.

Supplementary Table S4: Expression profile of heat shock protein family related genes in MCF-7-M2 derived cells

Gene symbol	Gene name	Lep	AAdipo-CM	CAF-CM
HSP90B1	Heat shock protein 90kDa beta, member 1	+2.38	+2.20	+2.7
HSP90AA1	Heat shock protein 90kDa alpha, class A member 1	+2.02	+1.76	+2.07
HSPH1	Heat shock 105kDa/110kDa protein 1	+2.02	+2.04	+1.88
HSPB8	Heat shock 22kDa protein 8	+1.80	+2.17	+1.71
HSPA1A	Heat shock 70kDa protein 1A	+2.74	+2.08	+2.25
HSPE1	Heat shock 10kDa protein 1	+1.26	ns	+1.58
HSPA4	Heat shock 70kDa protein 4	+1.30	+1.54	+1.48
HSPD1	Heat shock 60kDa protein 1 (chaperonin)	+1.39	+1.28	+1.33

Numbers represent the ratio of the values of treated compared with untreated MCF-7 M2 spheres obtained by microarray data analysis. Statistical significance was calculated with the Illumina DiffScore, only genes with a DiffScore ≤− 30 and ≥ 30, corresponding to a *p*-value of 0.001, were considered as statistically significant. ns: nonsignificant.
